# Extending the *PAX1* spectrum: a dominantly inherited variant causes oculo-auriculo-vertebral syndrome

**DOI:** 10.1038/s41431-022-01154-2

**Published:** 2022-07-25

**Authors:** Shannon Carter, Bridget J. Fellows, Kate Gibson, Louise S. Bicknell

**Affiliations:** 1 Genetic Health Service New Zealand, Christchurch Hospital, Christchurch, New Zealand; 2 Department of Biochemistry, University of Otago, Dunedin, New Zealand

**Keywords:** Medical genetics, Genetics research

## Abstract

Oculo-auriculo-vertebral syndrome (OAVS) is a clinically heterogeneous disorder, with both genetic and environmental contributors. Multiple genes have been associated with OAVS and common molecular pathways, such as retinoic acid and the PAX-SIX-EYA-DACH (PSED) network, are being implicated in the disease pathophysiology. Biallelic homozygous nonsense or hypomorphic missense mutations in *PAX1* cause otofaciocervical syndrome type 2 (OTFCS2), a similar but more severe multi-system disorder that can be accompanied by severe combined immunodeficiency due to thymic aplasia. Here we have identified a multi-generational family with mild features of OAVS segregating a heterozygous frameshift in *PAX1*. The four base duplication is expected to result in nonsense-mediated decay, and therefore cause a null allele. While there was full penetrance of the variant, expressivity of facial and ear features were variable. Our findings indicate there can be monoallelic and biallelic disorders associated with *PAX1*, and further implicate the PSED network in OAVS.

## Introduction

Disorders of the first and second branchial arches include Treacher Collins syndrome (TCS; MIM 154500), auricocondylar syndrome (ACS; MIM 602483), mandibulofacial dysostosis, Guion-Almeida type (MIM 610536), branchio-oto-renal syndrome (BOR; MIM 113650, 610896), Stickler syndrome (MIM 108300) and hemifacial microsomia/oculo-auriculo-vertebral spectrum (HFM/OAVS; MIM 164210). These disorders are thought to result from a combination of inadequate migration and formation of facial mesenchyme during early embryonic life [1]. In TCS, ACS, BOR and Stickler syndrome molecular pathways have been established, however for the spectrum of disease encompassed by OAVS, the full molecular etiology of the condition is the subject of ongoing investigation.

OAVS, also known as Goldenhar syndrome or hemifacial/craniofacial microsomia, is a heterogeneous and complex group of disorders [2]. Craniofacial appearances range from subtle facial asymmetry to HFM or orofacial clefts, preauricular skin tags to microtia and anotia, and epibulbar dermoids to micropthalmia and coloboma [3]. Extracranial defects of the cardiac, renal, vertebral and central nervous systems are reported [3–5]. This marked phenotypic variability and a lack of consensus on minimum diagnostic criteria has resulted in considerable discrepancy in reported prevalence, but it is generally now accepted at 1 in 5600 [4].

The etiology of OAVS is still largely unknown and felt to be multifactorial due to both environmental (maternal diabetes, antenatal exposure to vasoactive medications, smoking and twinning) and genetic factors during early embryogenesis [4]. With the widespread adoption of exome or genome sequencing, several genes have now been associated with OAVS [6–11], although many have only been identified through segregation in multi-generational single families. Most recently, a large exome/genome sequencing effort of trios identified haploinsufficient variants in *SF3B2* in multiple families segregating OAVS [12]. Variants were identified in 3% of sporadic and 25% of familial cases, indicating that while *SF3B2* genetic alterations are the most common cause of OAVS identified to date, there is wide genetic heterogeneity for this condition.

The PAX-SIX-EYA-DACH (PSED) network is involved in a variety of developmental processes including roles in morphogenesis of the branchial arches, which acts as the developmental basis for many of the clinical features of OAVS. A proposed mechanism for OAVS is that altered signaling networks cause disrupted migration of cranial neural crest cells, which are essential for normal facial mesenchymal tissue development [4].

The *PAX* family encode nuclear transcription factors involved in embryogenesis in vertebrates, with five out of the nine *PAX* genes associated with congenital disorders in humans to date. Biallelic hypomorphic or loss of function variants in *PAX1* have been described in several families with otofaciocervical syndrome type 2 (OTFCS2; MIM 615560) [13–16]. In severe cases, patients can also have severe combined immune deficiency, caused by underdevelopment or absence of the thymus [13].

Here we describe a novel dominant *PAX1* variant segregating with full penetrance but variable expressivity in a family with OAVS. All family members are affected with mild but definitive features of OAVS including HFM, misshapen ears and preauricular pits. Imaging on the three available family members did not detect any vertebral anomalies. Our findings suggest a novel cause of OAVS and confirm that heterozygous variants in *PAX1* can cause a clinically overt genetic disorder.

## Subjects and methods

### Clinical presentation of family

**Fig. 1:**
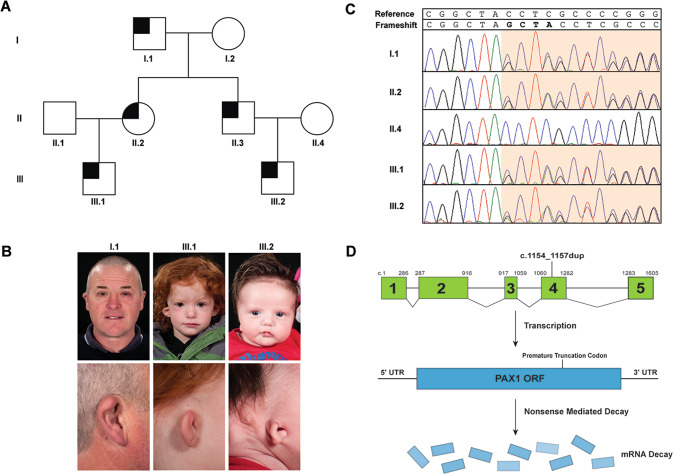
Clinical and genetic characterization of family. **A** Pedigree of OAVS features. **B** Facial features of affected family members with *PAX1* variant. III.1 and III.2 display right hemifacial microsomia and microtia, whilst I.1 has subtle facial asymmetry. Misshapen right ears are present in all three individuals ranging from over-folded ear in I.1 to the lobule-type ear in III.2. **C** Sanger sequencing of *PAX1*c.1154_1157dup variant. Heterozygous duplication results in overlapping peaks from the frameshift variant site (orange). **D** *PAX1*c.1154_1157dup variant located in Exon 4 generates a premature truncation codon in the mRNA transcript (p.Tyr386*) and likely stimulates degradation of the transcript by nonsense-mediated decay (NMD); therefore no protein would be produced from this allele.

We present a three-generation pedigree of five individuals with HFM and microtia in keeping with the OAVS spectrum (Table 1). The proband, III.1, presented at age 5 months with right sided HFM, microtia, small superior sinus and right sided conductive hearing loss. Subsequently the family pedigree revealed an affected mother (II.2), maternal grandfather (I.1), maternal uncle (II.3) and cousin (III.2) (Fig. 1A).

**Table 1:** Clinical summary of family compared to typical features of OTFCS2.

Clinical features	This family					OTFCS2
	I.1	II.2	II.3	III.1	III.2	
Hemifacial microsomia	+ (subtle)	+	+ (subtle)	+	+	+
Microtia	+	+	+	+	+	+
Hearing loss	+	na	na	+	+	+
Preauricular pits	+	+	na	+	−	+
Misshapen ears	+	−	na	+	+	+
Intellectual disability/developmental delay	−	+ (mild)	+ (mild)	−	−	+
Shoulder girdle anomalies	−	na	na	−	−	+
Vertebral anomalies	−	na	na	−	−	+

*na* not available.

Following a normal antenatal course, III.1 was born at term with a birth weight on the 75th centile and was diagnosed with right microtia with a well-formed lobe, residual concha and a small superior sinus at birth (Fig. 1B). The left ear had a cupped and over-folded appearance. Musculoskeletal, cardiovascular and ophthalmological examinations were unremarkable without epibulbar dermoids or vertebral anomalies.

As III.1 grew, a facial asymmetry was noted in keeping with a diagnosis of right hemifacial microtia. He had a normal appearance of his mandible with class I malocclusion. Imaging by CT confirmed an aberrant facial nerve course along with hypoplastic right external auditory canal and middle ear with a dysplastic appearance of his right malleus and incus and an absent stapes. III.1 has moderately severe conductive hearing loss on the right. III.1 attends mainstream school and has no history of developmental delay or intellectual disability. X-ray imaging of the spine revealed no vertebral anomalies.

III.2 was born to non-consanguineous parents after a healthy pregnancy at term with a birth weight on the third centile. III.2 was diagnosed with grade III microtia, lobule-type ear and noted to have a rightward deviated chin and immediately appreciable facial asymmetry in this instance (Fig. 1B). Again musculoskeletal, cardiovascular and ophthalmological examinations were unremarkable. III.2 also had class I malocclusion. III.2 was troubled by a recurrently discharging postauricular sinus throughout his infancy and early childhood. Imaging confirmed a narrow and duplicated internal ear canal accounting for this discharging sinus. In addition III.2 had an absent stapes, and connection to the inner ear. His mastoid was hypoplastic, however appearances of the cochlear and inner ear structures were normal. III.2 also had moderately severe hearing loss on the right. Although III.2 had mildly delayed expressive language this had normalized on school entry with speech and language support and there is no evidence of developmental delay or intellectual disability. X-ray imaging of the spine revealed no vertebral anomalies.

At the initial consultation with III.1 we met I.1 and II.2 who both had subtle facial asymmetry. I.1 had an over-folded right ear and was otherwise well with no significant musculoskeletal, cardiovascular or ophthalmological history, and normal vertebrae on X-ray imaging. II.1 was noted to have a right preauricular pit only and similarly was otherwise well. II.3 has not been formally assessed by our service but is reported to have “small ears and petite facial features”.

## Methods

Five members of this family (I.1, II.2, II.4, III.1, III.2) were subjected to exome sequencing using the Agilent V5 capture kit, followed by sequencing on an Illumina HiSeq 4000. Sequencing data were processed by a standard GATK-based pipeline as previously described [17]. Variants were prioritized based on an autosomal dominant inheritance model, starting with variants absent in gnomAD that were of high functional impact.

Sanger sequencing was undertaken to confirm the presence or absence of the *PAX1* variant in all family members. Amplification of DNA and sequencing was undertaken using oligonucleotides PAX1ex4F CGTCTCAGTGATGGCGTG and PAX1ex4R CCCAGTACCCTTCCTAACCC, using methodology previously described [17].

## Results

Prioritization of high impact variants heterozygous in all affected individuals highlighted a frameshift variant in *PAX1*; c.1154_1157dup, p.(Tyr386*) (RefSeq: NM_001257096.2), which was absent from gnomAD. Sanger sequencing confirmed segregation within the family (Fig. 1C). The position of the frameshift variant would predict that transcripts harboring this allele would undergo nonsense-mediated decay, and therefore be a null allele (Fig. 1D). This variant can be classified as pathogenic (PVS1, PM2, PP1) using ACMG/AMP criteria [18], although we note it can be difficult to accurately apply such criteria when both monoallelic and biallelic disorders are associated with the same gene, with variable expressivity and penetrance.

## Discussion

Biallelic *PAX1* mutations are associated with OTFCS2 [13–16], whereas this family segregates a heterozygous *PAX1* variant, with full penetrance but variable expressivity. In comparing the clinical features of this family with OTFCS2, there is phenotypic overlap with this family, of HFM and microtia. There was no evidence of significant developmental delay in this family, nor any vertebral anomalies in the three individuals available for imaging, both of which are observed in patients with biallelic *PAX1* variants [3].

A large consanguineous kindred was recently reported where patients were affected with OTFCS2 as well as severe combined immunodeficiency [13], caused by a homozygous nonsense mutation in *PAX1*. Interestingly, six family members who were heterozygous for the segregating variant were reported to have preauricular pits, although no other phenotypic information was provided. Combined with our findings, this suggests that heterozygous mutations in *PAX1* are capable of causing a clinically overt disorder with potentially variable penetrance and expressivity. It remains striking that we observed full penetrance of the duplication variant in this multi-generational family, even with more subtle features. Systematic clinically assessment of *PAX1* variant carrier individuals would be valuable to help clinically define the penetrance and expressivity of *PAX1* variants, in light of the family presented here.

There is an increasing number of reported genes associated with OAVS, as well as putative risk alleles from GWA studies [19]. A common theme occurring is the involvement of the PSED network, for example, a recurrent missense variant occurring in *EYA3* or intragenic genomic alterations of *DACH1* or *DACH2* [10, 20]. All of these are predicted to act through a dominant genetic mechanism, as opposed to the biallelic loss of function mechanism for *PAX1* in OTFCS2.

Many of the genes identified thus far have utilized the multi-generational segregation of OAVS to prioritize variants. Such familial examples also provide insight into the variable penetrance and expressivity of the OAVS phenotypes. As the genetic basis of familial OAVS expands, it will be interesting to learn whether OAVS subcategories could be classified using the observed spectrum of penetrance and expressivity, or on the basis of associated developmental pathways.

## Data Availability

Variant details are available on ClinVar, accession: SCV002098398.
